# Challenges in the Detection of Clinically Useful Biomarkers for the Diagnosis of Delirium in Older People in the Emergency Department—A Case–Control Study

**DOI:** 10.3390/life12081127

**Published:** 2022-07-27

**Authors:** Angela Soler-Sanchis, Francisco Miguel Martínez-Arnau, José Sánchez-Frutos, Pilar Pérez-Ros

**Affiliations:** 1 Department of Nursing, Faculty of Nursing and Podiatry, Universitat de València, 46010 Valencia, Spain; angela.soler@uv.es (A.S.-S.); maria.p.perez-ros@uv.es (P.P.-R.); 2 Hospital Francesc de Borja, Departament de Gandia, 46702 Gandia, Spain; 3 Department of Physiotherapy, Universitat de València, Gascó Oliag 5, 46010 Valencia, Spain; jose.sanchez-frutos@uv.es; 4 Frailty and Cognitive Impairment Research Group (FROG), Universitat de València, Menendez y Pelayo 19, 46010 Valencia, Spain

**Keywords:** biomarkers, delirium, older, emergency department, challenge

## Abstract

Background: The identification of biomarkers associated with delirium in the emergency department could contribute to the understanding, prediction and diagnosis of this disorder. The present study was carried out to identify biomarkers included in easily and quickly obtained standard blood examinations in older patients with delirium in the emergency department. Methods: A case–control study was carried out in the emergency department of Francesc de Borja Hospital (Gandía, Valencia, Spain). Older adults (≥65 years of age) diagnosed with delirium (*n* = 128) were included. Cases due to alcohol or substance abuse were excluded. Controls were selected on a randomized basis from the remaining patients (*n* = 128). All laboratory test parameters included in the routine blood and urine tests of the emergency department were collected. Results: The mean age of the patients was 81.24 ± 7.51 years, and 56.2% were males, while the mean age of the controls was 78.97 ± 7.99 years, and 45.3% were males. Significant differences were found between the cases and controls in relation to the following parameters: urea 43 (32–58) mg/dL versus 50 (37–66) mg/dL, respectively; neutrophils 69.6 (62.05–78.75)% versus 75.5 (65.1–83.2)%; monocytes 8.7 (7–10.4)% versus 7.6 (5.5–9.2)%; platelets 213 (159–266) × 10^9^/L versus 224 (182–289) × 10^9^/L; neutrophil–lymphocyte ratio 3.88 (2.45–7.07) versus 5 (2.75–8.83); platelet–lymphocyte ratio 281.4 (210–360) versus 357.1 (257.8–457.1); and mean platelet volume 10.6 (10–11.5) fl versus 10.4 (9.67–10.9) fl. Although the mean values were above desirable levels in both groups, they were higher for most parameters in the control group. No significant differences were observed in C-reactive protein concentration (9.99 (1.69–51) mg/L versus 12.3 (3.09–65.97) mg/L). Conclusions: The identification of delirium biomarkers poses difficulties due to the urgent nature of the disorders found in older people admitted to the emergency department. Research in this field is needed, since it would allow early identification and treatment of delirium.

## 1. Introduction

Delirium is a severe neuropsychiatric syndrome [1], defined as acute and fluctuating changes in patient cognition and consciousness [2]. It affects up to 80% of all acute-care patients, and particularly the older population [3]. Early recognition of the condition is crucial in order to start effective treatment and minimize the serious consequences related to loss of function and quality of life, and increased dependency [1].

Delirium in the emergency department (ED) is associated with a longer hospital stay, functional and cognitive impairment, an increased risk of falls and a greater likelihood of institutionalization, ultimately with an increase in patient morbidity and mortality [4,5].

In recent years, the detection of delirium in the ED has become a high-performance research objective, since it is an underdiagnosed disorder [6]. Studies indicate that 75% of all cases of delirium go unidentified in the ED [5], and this problem is expected to increase in the coming years as a result of aging of the population and the growing number of older patients seen in the ED [7].

In addition to knowledge of the predisposing and triggering risk factors, comorbidities and drug therapies, alterations of certain blood parameters could contribute to the recognition of delirium [1]. Different authors have therefore proposed potential biomarkers such as S100β and cortisol, which may help develop new therapies and monitor the response to treatment, establish an early diagnosis, assess severity, and define endpoints for the resolution of delirium [3].

There is little evidence in the literature on the clinical use of any delirium biomarkers, though some markers such as S-100 beta, insulin-like growth factor-1, cortisol and C-reactive protein (CRP) have shown some promising results [2,3]. The literature concludes that no specific biomarkers afford complete certainty in the early diagnosis of delirium, though it has been postulated that all inflammatory biomarkers could be related to delirium, with the exception of IL-1β and IL-12. A review of possible markers that could be included in standard blood tests which can be easily and quickly performed in the hospital ED identified albumin, creatinine, cholinesterase, CRP, neutrophil–lymphocyte ratio (NLR) and the leukocyte, red cell or platelet series as potential candidates [8].

The lack of studies on the prevalence of delirium and its detection rates in the ED [6] make the identification of biomarkers associated with delirium necessary, since such markers could contribute to the understanding, prediction and diagnosis of delirium [9]. Clinically useful biomarkers are needed in the ED due to the idiosyncrasies of urgent patient care. The aim of the present study was to determine whether there are clinically useful biomarkers recorded in the ED for application to older people with delirium.

## 2. Materials and Methods

### 2.1. Study Design and Setting

A retrospective, unpaired case–control study was conducted to establish possible biomarkers for the detection of delirium in older persons (≥65 years of age) admitted to the ED of Francesc de Borja Hospital (Gandía, Valencia, Spain)—a secondary, academic hospital serving a population of 188,000 inhabitants. The study has followed the STROBE guidelines for reporting in observational studies (Supplementary File S1).

### 2.2. Participants

The patients included in this study were aged 65 years or older and reported to the ED between 1 January and 31 December 2020. Cases were subjects coded according to the International Classification of Diseases—Ninth Revision (ICD-9) with delirium in the ED (code R41. 0 for Disorientation and F05—Delirium due to known physiological condition) as either a primary or secondary diagnosis recorded in the electronic medical record. The delirium indicator variable was obtained by the treating physician based on the DSM-V criteria [10]. Cases of alcohol- or substance-induced delirium were excluded from this study. A previous study in this population analyzed risk factors and information regarding the characteristics of the population in terms of emergency triage [11].

Once the cases were identified, the controls were randomly selected from the remaining patients meeting the inclusion criteria through computer-based block randomization using the XLstat^®^ tool.

### 2.3. Sample Size

A representative sample of 117 subjects per group was calculated for the unpaired case–control study, assuming an expected proportion of delirium in the patient group of 9% versus 1% among the cases, for an alpha error of 5% and a statistical power of 80%.

### 2.4. Data Collection

All data were collected from the electronic medical records: sociodemographic data, age and sex, and the number of drugs prescribed together with the main pharmacotherapeutic groups related to delirium [12]. The medical diagnosis after discharge from the ED was coded by systems according to the main problems (cardiac, respiratory, neurological, renal, trauma, hematological, digestive, stroke, COVID-19, anxiety, fever and sepsis). In cases of delirium, the secondary diagnosis reflected in the medical records was analyzed as the organic cause of the onset of delirium, following the same grouping as previously indicated.

All laboratory test parameters included in the routine blood tests of the ED were collected. Biochemistry: glucose, urea, creatinine, sodium, potassium, chloride, total bilirubin, amylase, GOT/AST, GPT/ALT, cholinesterase and C-reactive protein (CRP). Blood count: erythrocytes, hemoglobin, hematocrit, mean corpuscular volume (MCV), mean corpuscular hemoglobin (MCH), mean corpuscular hemoglobin concentration (MCHC), red cell distribution width (RDW), leukocytes, neutrophils, lymphocytes, monocytes, eosinophils and basophils (as percentage and absolute values), platelets, and neutrophil–lymphocyte ratio (NLR) and platelet–lymphocyte ratio (PLR). Mean platelet volume (MPV), prothrombin time, Quick index, international normalized ratio (INR), activated partial thromboplastin time (aPTT), thromboplastin time ratio and fibrinogen, as variables related to coagulation, were also recorded. Finally, data related to urinalysis were included urine density, urine pH, urine protein, urine glucose, urine ketones, urine bilirubin, urobilinogen, urine erythrocytes and urine leukocytes. All blood and urine tests were performed during the course of the episode in the Emergency Department. The exact time of onset of delirium is unknown, but in most cases, subjects present within hours of symptoms onset, and at most within the first 12–24 h.

### 2.5. Ethical Considerations and Data Confidentiality

This study was approved by the Ethics Committee of the Hospital. The data obtained were kept confidential in accordance with Spanish legislation on personal data protection (Ley Orgánica 3/2018, of 5 December). This study was conducted in accordance with the principles of the Declaration of Helsinki.

### 2.6. Statistical Analysis

Descriptive statistics were computed to characterize the sample and data distribution, and to check assumptions. The variables were reported as proportions and/or means and standard deviation (SD). The Kolmogorov–Smirnov test was used to assess normal data distribution. Parametric (Student *t* test) and non-parametric testing (Mann–Whitney U test) were used to compare quantitative variables, while the chi-square test was used for categorical variables. Two-factor between-subjects models were generated to analyze specific biomarker scores by groups (cases and controls), according to diagnostic groups. In order to adjust the *p*-value for multiple comparisons, Bonferroni corrections were used with a significance level of *p* < 0.003. All other statistical significance was reported at *p* < 0.05.

The study data were entered in MS Excel spreadsheets, and statistical processing was carried out using the SPSS version 23.0 statistical package (IBM Corp., Armonk, NY, USA).

## 3. Results

A total of 128 patients and 128 controls were included in this study. The mean age was higher in the male-dominated patient group than in the control group. The median number of prescribed daily drugs was also higher among the patients (Table 1). A significantly higher proportion of psychotropic and anesthetic prescriptions was recorded among the patients versus the controls.

**Figure 1:**
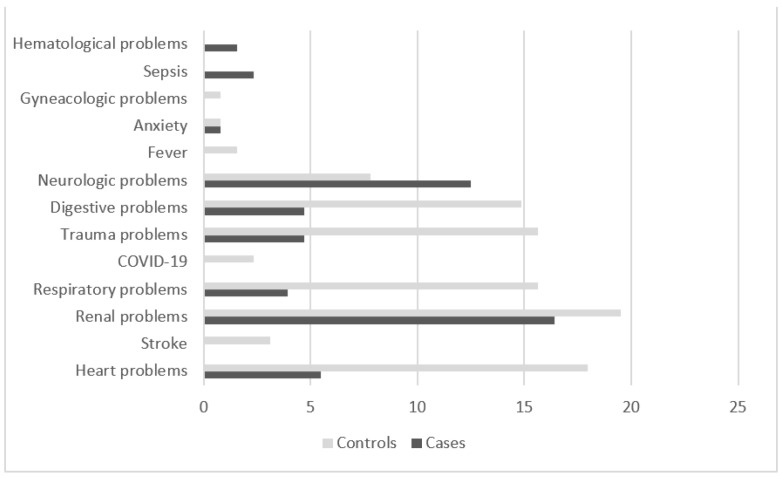
Distribution of the main diagnoses in the controls and of the secondary diagnoses in the patients (cases).

On analyzing the medical diagnoses at discharge among the controls, most of the diagnoses were seen to correspond to renal, cardiac, respiratory and trauma problems. Among the patients, secondary diagnoses were identified in 52.35% of the cases (*n* = 67) and thus reflected probable organic causes of delirium. On the other hand, in 47.65% of the patients (*n* = 61), no cause of delirium was detected, and no secondary diagnoses were recorded in the medical history. In those cases where a secondary medical diagnosis was recorded, we observed a greater percentage of patients with neurological disorders (mainly dementia), and renal and cardiac problems (Figure 1).

Statistically significant differences were observed on analyzing the differences in blood and urine parameters between the two groups, with lower values corresponding to urea, neutrophils%, platelets, NLR and PLR among the patients than in the control group. On the other hand, higher values were observed corresponding to monocytes% and MPV. A group analysis of the normality ranges of each parameter was performed, and statistically significant differences were found in the same parameters (Table 2 and Table 3)

The analysis of the parameters that showed significant differences by groups in the two-factor model according to the diagnoses grouped by areas (Table 4) revealed only differences in relation to CRP in respiratory disorders, with higher values among the controls.

To assess possible interactions of the prescribed drugs at the level of the laboratory test markers, we analyzed a two-factor model involving the pharmacotherapeutic groups that showed differences in the bivariate model: psychotropic agents and anesthetics (Table 1)—no statistically significant differences were found for either psychotropic agents (*p* = 0.058) or anesthetics (*p* = 0.130) (Table 4).

## 4. Discussion

The increase in the number of hospital emergency department (ED) visits by older people is a worldwide phenomenon [13,14,15]. Furthermore, the figures will further grow over the coming years as the older population continues to increase. Delirium is an under-diagnosed and under-treated syndrome in all settings, reaching a prevalence of up to 38% in the ED [4]. The prevention, detection and treatment of delirium is a challenge nowadays, due to its multifactorial nature, in addition to the presence of subtypes with different presentations and symptoms, as well as the concurrence of delirium with other patient comorbidities [16]. Our aim was to identify possible useful biomarkers for the detection of delirium in older people in the ED, and although the participants showed out-of-range mean values for urea, neutrophils%, monocytes%, platelets, NLR, PLR and mean platelet volume, no biomarker was identified.

The detection of delirium is currently mainly based on the DSM-V diagnostic criteria, without the use of any specific biomarker. The scientific community is calling for the search for biomarkers that could help in the understanding and diagnosis of this syndrome [17]. The pathophysiology of delirium has several hypotheses, with an inflammatory mechanism being the most solid proposal. An organic cause would provoke an inflammatory process affecting the central nervous system. In this acute process, cytokines and systemic mediators are released into the bloodstream. Together with several events such as activation of the vagus nerve, the choroid plexus, activation of the coagulation pathways and impairment of the microcirculation, these systemic alterations could disrupt the blood–brain barrier leading to a central nervous system inflammatory cascade [18].

Based on this hypothesis, there are biomarkers in different fluids such as cerebrospinal fluid or blood that are useful for research, including cytokines, enzymes, growth factors and hormones, or which are useful in clinical practice, such as proteins, creatinine, NLR or PLR. Research in recent years has identified biomarkers of delirium in older people with acute and critical disease conditions, requiring differentiation among surgical, medical and critical patients; such biomarkers are difficult to apply in daily practice, and are not entirely consistent [8,19].

An ideal biomarker should be easily identifiable, cost-effective, reliable to allow targeted therapy, and at the same time should be highly sensitive and very specific. To our knowledge, no studies have evaluated biomarkers with these characteristics in the emergency care setting [20].

Our results did not reveal any particular biomarker, and this may be due to several factors. A first factor is the nature of the ED itself, with the patients who attend being those requiring rapid care in the face of a recently established or aggravated disorder. A second factor is that a potential organic cause of delirium cannot be identified in the ED in all cases, and it is in later days during hospital admission or at home when the required information may be obtained—which also makes it difficult to interpret the analytical results. Lastly, the laboratory test data are obtained at the time of ED attendance, and at this point the new onset condition might not yet have modified the possible biomarkers in the biological samples. In this regard, postsurgical studies are characterized by serial pre- and postsurgical laboratory tests, while studies conducted in the medical setting typically analyze blood samples obtained from the first 24 h of admission onwards [2,21].

In standard blood tests, the included biomarkers are albumin and creatinine, and may be useful in identifying an increased risk of delirium [8], since low albumin levels appear to be associated with an increased risk of delirium in postsurgical patients [22]. Similarly, low creatinine levels have been identified as a risk factor in a cohort of heart surgery patients [23]—though it should be noted that these are not specific targets for delirium prevention, as they are routinely addressed with respect to other clinical issues. Urea has also been described as a possible altered marker in cardiac patients [21]. In our study, we likewise observed higher urea values in patients with delirium and a secondary diagnosis of heart disease.

In contrast to the above, C-reactive protein (CRP) is considered to be a clinically useful biomarker of delirium in both medical and surgical patients [19,20,24]. Several authors have detected higher CRP levels in older or very older people presenting delirium than in non-delirious patients [24,25,26]. This marker could even be of help as a diagnostic tool to facilitate the identification of hypoactive delirium. According to our results, delirious patients have lower mean concentrations than controls, though without reaching statistical significance. This may be related to the fact that CRP is elevated as an inflammatory response [27], and our entire patient sample was in an acute and probably recently established phase of disease at the time of admission to the ED. In addition, in our case, the laboratory tests were performed at the time of attendance, whereas in other studies they were performed 24 h after admission [28]. On the other hand, it would also be possible to analyze these differences according to the probable organic cause underlying delirium. In our study, higher CRP values were found in patients with cardiac problems, in coincidence with the observations of other studies in which high CRP values were related to delirium in older people with acute disease and post operative delirium in heart surgery [28,29].

We recorded no differences in cholinesterase values, in concordance with the findings of other authors [27,30]. This may be due to the very specific character of cholinesterase as a marker of liver synthesis function.

Interest in the neutrophil–lymphocyte ratio (NLR) has been on the rise ever since differences were found [28] in older people on obtaining laboratory test data 24 h after admission, with NLR values of 9.1 in the group of older people with delirium. Our results yielded figures of 5.53 ± 4.25 in older people with delirium, as opposed to non-delirium with 7.55 ± 8.35. The urgent nature of care in the ED could determine that this alteration in NLR is high in both groups and higher than the reported cut-off value of 3.626 [31], with a sensitivity of 75.2% and a specificity of 63.4% in older hospitalized people after 24 h of admission. Other authors have also analyzed NLR, without finding differences [32] after the first 24 h of stay in the Intensive Care Unit or in specific disease contexts such as cardiac surgery, where differences in NLR were only found three days after surgery [20]. As we can see, knowing the comorbidity of the patients, their current medications, and the possible acute or organic cause, would facilitate interpretation of the results and their comparison. In any case, our sample of older people with delirium showed no higher values for any of the causes analyzed, except neurological disease or anxiety. 

Platelets count and PLR looks like it could also be a marker of delirium [32]. Our results are also similar to those previously described with the NLR. Cases have lower platelet count and PLR than controls, but the mean of cases is higher than those reported by other authors [32,33]. It is important to analyze the separate pathologies that could be the cause of the onset of delirium in order to be able to discern a little about these aspects.

Significant diversity is currently found in the literature regarding the type of study design, the biomarkers examined, the patient populations and the settings in which research is conducted (medical, surgical [from cardiac to orthopedic surgery], mixed or even undefined). It can be affirmed that until properly designed studies become available, it is unlikely for altered biomarker levels to be of help in clinical practice [8].

To our knowledge, this is the first article to provide all the clinical analytical data obtained in the ED. Despite not finding a possible biomarker, our results will allow researchers to compare different cohorts and analyze according to pathologies. The limitations of the present study include those inherent to its retrospective design, which precludes the analysis of other possible blood biomarkers in the sample analyzed. Likewise, it was not possible to monitor laboratory test parameters beyond the first 24 h of admission, when greater biomarker alterations could perhaps be observed—though the values then might also be biased as a result of the treatments administered. The race of the participants in this study was also not analyzed. Although the majority were Caucasian, these data are not available. Lastly, there is little literature on biomarkers of delirium in older people in the ED, which makes it difficult to compare results for patients of this kind.

## 5. Conclusions

Older patients with delirium admitted to the hospital emergency department showed out-of-range mean values in urea, neutrophils%, monocytes%, platelets, NLR, PLR, and mean platelet volume. Despite this, no biomarker was identified, due to the high values also found in the control group. There is difficulty in identifying inflammatory biomarkers of delirium in the ED, due to the urgent nature of the disorders mainly seen in the hospital ED setting.

## Figures and Tables

**Table 1:** Characteristics of the sample and prescribed drugs.

Variable	*n*	Case	*n*	Control	*p*-Value
Age in years, mean (SD)	128	81.24 (7.51)	128	78.97 (7.99)	0.02 ^t^
Sex					
Female, %	56	43.8	70	54.7	0.08 ^χ2^
Male, %	72	56.2	58	45.3	
Prescribed daily drugs, mean (SD)	128	8 (5–11)	128	7 (4–9)	0.038 ^t^
Opioids, %	14	43.8	18	56.3	0.450
Psychotropic drugs, %	83	64.3	46	35.7	<0.001
Oral antidiabetic drugs, %	30	60.0	20	40.0	0.115
Antiarrhythmic agents, %	30	60.0	20	40.0	0.115
Analgesics, %	59	45.4	71	54.6	0.134
Anticholinergic agents, %	30	60.0	20	40.0	0.115
Anesthetics, %	23	76.7	7	23.3	0.002
Antiplatelet drugs, %	22	46.8	25	53.2	0.628
Corticosteroids, %	41	56.2	32	43.8	0.213
Diuretics, %	37	50.7	36	49.3	0.890
Thyroid agents, %	10	41.7	14	58.3	0.391
Insulin, %	15	62.5	9	37.5	0.198

^t^: Student *t* test; ^χ2^: chi-square test.

**Table 2:** Total sample and case–control blood and urine biomarker values.

Biochemistry	N	Total Median (IQ Range)	N	Case Median (IQ Range)	N	Control Median (IQ Range)	*p*-Value
Glucose, mg/dL	222	118 (104–150.25)	127	119 (104–158)	95	118 (106–145)	0.915 ^u^
Urea, mg/dL	212	45 (33–63)	123	43 (32–58)	89	50 (37–66)	0.006 ^u^
Creatinine, mg/dL	222	0.99 (0.82–1.34)	127	0.95 (0.77–1.27)	95	1.06 (0.85–1.41)	0.079 ^u^
Sodium, mmol/L	222	140 (138–142)	127	140 (138–142)	95	140 (137–142)	0.926 ^u^
Potassium, mmol/L	201	4.35 (3.99–4.63)	113	4.32 (3.93–4.56)	88	4.38 (4.01–4.64)	0.350 ^u^
Chlorine, mmol/L	222	101 (98–103)	127	100 (98–103)	95	101 (99–103)	0.348 ^u^
Total bilirubin, mg/dL	137	0.48 (0.35–0.82)	83	0.46 (0.35–0.81)	54	0.5 (0.34–0.87)	0.809 ^u^
Amylase, U/L	109	56 (37.5–82)	67	52 (37–76)	42	62.5 (40.5–89.25)	0.197 ^u^
GOT/AST, U/L	100	20.5 (17–27)	61	21 (17–26.5)	39	20 (16–28)	0.915 ^u^
GPT/ALT, U/L	194	15 (11–21)	115	15 (11–22)	79	14 (12–21)	0.979 ^u^
Cholinesterase, U/L	44	5883 (4640.75–7626)	22	5898 (5385.5–8290.5)	22	5837.5 (4473–7211.25)	0.425 ^u^
C-reactive protein, mg/L	211	11.04 (2.28–51.48)	123	9.99 (1.69–51)	88	12.3 (3.09–65.97)	0.369 ^u^
Blood count							
Erythrocytes, ×10^12^/L	222	4.43 (3.97–4.82)	127	4.43 (3.95–4.85)	95	4.38 (3.99–4.8)	0.521 ^t^
Hemoglobin, g/dL	222	13.2 (11.9–14.4)	127	13.2 (11.9–14.4)	95	13.2 (11.9–14.3)	0.215 ^t^
Hematocrit, g/dL	222	39.6 (35.88–43.33)	127	39.6 (35.6–43.5)	95	39.8 (36.1–42.9)	0.651 ^t^
Mean corpuscular volume, fl	220	90.3 (87.02–94.03)	126	90.3 (86.97–94.32)	94	90.25 (87.07–93.72)	0.815 ^u^
Mean corpuscular hemoglobin, pg	218	30.2 (28.8–31.7)	125	30.4 (28.75–31.7)	93	29.8 (28.8–31.6)	0.370 ^u^
Mean corpuscular hemoglobin concentration, %	214	33.4 (32.5–34.1)	123	33.4 (32.4–34.2)	91	33.2 (32.5–34)	0.298 ^u^
Red cell distribution width, %	213	13.6 (12.9–14.75)	122	13.55 (12.8–14.6)	91	13.8 (13–15)	0.255 ^u^
Leukocytes, ×10^9^/L	222	8.9 (7–11.73)	127	8.8 (7.1–11.7)	95	9.5 (6.9–12.4)	0.558 ^u^
Neutrophils, %	216	71.45 (62.85–80.76)	125	69.6 (62.05–78.75)	91	75.5 (65.1–83.2)	0.005 ^t^
Lymphocytes, %	216	15.85 (10.03–24.5)	125	17.4 (10.75–25)	91	15 (8.9–23.5)	0.292 ^t^
Monocytes, %	215	8.2 (6.8–9.9)	124	8.7 (7–10.4)	91	7.6 (5.5–9.2)	0.002 ^t^
Eosinophils, %	215	0.8 (0.3–2)	124	0.9 (0.3–2.45)	91	0.6 (0.2–1.8)	0.333 ^u^
Basophils, %	215	0.4 (0.2–0.6)	124	0.4 (0.2–0.6)	91	0.4 (0.3–0.6)	0.969 ^u^
Neutrophils, ×10^9^/L	221	6.1 (4.6–9.1)	126	5.9 (4.6–8.55)	95	6.6 (4.5–9.2)	0.282 ^u^
Lymphocytes, ×10^9^/L	221	1.5 (1–2)	126	1.5 (1–2)	95	1.4 (0.8–2)	0.192 ^u^
Monocytes, ×10^9^/L	222	0.7 (0.5–1)	127	0.7 (0.6–1)	95	0.7 (0.5–0.9)	0.084 ^u^
Eosinophils, ×10^9^/L	222	0.1 (0.0–0.2)	127	0.1 (0.0–0.2)	95	0.1 (0–0.2)	0.225 ^u^
Basophils, ×10^9^/L	222	0.0 (0.0–0.1)	127	0.0 (0–0.1)	95	0.0 (0–0.1)	0.105 ^u^
Platelets, ×10^9^/L	222	218 (174.25–278.25)	127	213 (159–266)	95	224 (182–289)	0.028 ^t^
Neutrophil–lymphocyte ratio	221	4.45 (2.55–7.55)	126	3.88 (2.45–7.07)	95	5 (2.75–8.83)	0.02 ^t^
Platelet–lymphocyte ratio	222	301.5 (219.5–410)	127	281.4 (210–360)	95	357.1 (257.8–457.1)	0.001 ^t^
Mean platelet volume, fl	204	10.5 (9.9–11.18)	118	10.6 (10–11.5)	86	10.4 (9.67–10.9)	0.009 ^t^
Coagulation							
Prothrombin time, seconds	211	14.3 (13.3–16.7)	124	14.25 (13.15–16.67)	87	14.4 (13.4–16.7)	0.551 ^u^
Quick index, %	212	88.5 (71–99.75)	124	89.5 (71–100)	88	88 (70.25–98)	0.446 ^u^
INR	211	1.08 (1–1.27)	124	1.07 (1–1.27)	87	1.09 (1.01–1.28)	0.360 ^u^
Activated partial thromboplastin time, seconds	198	29.8 (27.4–34.2)	117	30.01 (27.1–34.25)	81	29.6 (27.9–33.2)	0.945 ^u^
Thromboplastin time ratio	198	1 (0.91–1.12)	117	1 (0.9–1.13)	81	0.99 (0.93–1.12)	0.918 ^u^
Fibrinogen, mg/dL	142	468.5 (371.75–579.25)	84	469 (371.25–579.25)	58	459.5 (375–581)	0.908 ^t^
Urine							
Density, mg/ml	140	1016 (1012.25–1022)	104	1016 (1013–1023)	36	1016 (1011.25–1021)	0.490 ^u^
pH	140	6 (5–7)	104	6 (5–7)	36	5 (5–6)	0.175 ^u^
Urine proteins, mg/dL	140	25 (0.0–75)	104	25 (0.0–75)	36	12.5 (0.0–75)	0.986 ^u^
Urine glucose, mg/dL	140	0.0 (0–0)	104	0.0 (0–0)	36	0.0 (0–0)	0.816 ^u^
Urine ketones, mg/dL	140	0.0 (0–0)	104	0.0 (0–3.75)	36	0.0 (0–0)	0.162 ^u^
Urine bilirubin, mg/dL	139	0.0 (0–0)	103	0.0 (0–1)	36	0.0 (0–0)	0.699 ^u^
Urobilinogen, mg/dL	140	0.0 (0–0)	104	0.0 (0–0)	36	0.0 (0–0)	0.279 ^u^
Urine erythrocytes, cells/µL	140	10 (0.0–25)	104	10 (0.0–25)	36	25 (0.0–50)	0.243 ^u^
Urine leukocytes, cells/µL	140	0.0 (0–100)	104	0.0 (0–100)	36	25 (0.0–400)	0.092 ^u^

^t^: Student *t* test; ^u^: Mann–Whitney U test.

**Table 3:** Total sample and case–control blood and urine biomarker values in desired ranges.

		Case	Control	*p*-Value
Biochemistry	Ranges	%	%	
Glucose	Below desirable (≤69 mg/dL)	100	0	0.646
	Desirable (70–100 mg/dL)	58.4	41.6	
	Above desirable (≥101 mg/dL)	56.1	43.9	
Urea	Below desirable (≤16.9 mg/dL)	100	0	0.06
	Desirable (17–50 mg/dL)	63.6	36.4	
	Above desirable (≥ 50.1 mg/dL)	48.1	51.9	
Creatinine	Below desirable (≤0.49 mg/dL)	75	25	0.272
	Desirable (0.50–0.90 mg/dL)	63.3	36.7	
	Above desirable (≥0.91 mg/dL)	53.2	46.8	
Sodium	Below desirable (≤134.9 mmol/L)	65.2	34.8	0.58
	Desirable (135–145 mmol/L)	55.8	44.2	
	Above desirable (≥145.1 mmol/L)	66.7	33.3	
Potassium	Below desirable (≤3.49 mmol/L)	69.2	30.8	0.583
	Desirable (3.5–5.5 mmol/L)	55.6	44.4	
	Above desirable (≥5.51 mmol/L)	50	50	
Chlorine	Below desirable (≤94.9 mmol/L)	68.6	31.3	0.615
	Desirable (95–115 mmol/L)	56.4	43.6	
	Above desirable (≥115.1 mmol/L)	50	50	
Bilirubin	Desirable (0.1–1 mg/dL)	62.3	37.7	0.029
	Above desirable (≥1.1 mg/dL)	0	100	
Amylase	Below desirable (≤4.99 U/L)	100	0	0.084
	Desirable (5–120 U/L)	62.7	37.3	
	Above desirable (≥120.1 U/L)	20	80	
GOT/AST	Below desirable (≤1.9 U/L)	-	-	0.68
	Desirable (2–40 U/L)	59.7	40.3	
	Above desirable (≥40.1 U/L)	53.8	46.2	
GPT/ALT	Below desirable (≤1.9 U/L)	-	-	0.405
	Desirable (2–37 U/L)	62.5	37.5	
	Above desirable (≥37.1 U/L)	50	50	
Cholinesterase	Below desirable (≤3899.9 U/L)	40	60	0.521
	Desirable (3900–13,200 U/L)	52.6	47.4	
	Above desirable (≥13,200 U/L)	0	100	
C-reactive protein	Desirable (0.0–6.00 mg/L)	58.8	41.3	0.844
	Above desirable (≥6.1 mg/L)	57.4	42.6	
Blood Count				
Erythrocytes	Below desirable (≤3.49 × 10^12^/L)	61.9	38.1	0.654
	Desirable (3.5–5.5 ×10^12^/L)	56.2	43.8	
	Above desirable (≥5.51 × 10^12^/L)	71.4	28.6	
Hemoglobin	Below desirable (≤11.99 g/dL)	58.6	41.4	0.955
	Desirable (12–15.5 g/dL)	56.9	43.1	
	Above desirable (≥15.51 g/dL)	55	45	
Hematocrit	Below desirable (≤11.99 g/dL)	57.8	42.2	0.748
	Desirable (12–15.5 g/dL)	55.9	44.1	
	Above desirable (≥15.51 g/dL)	64	36	
Mean corpuscular volume	Below desirable (≤81.99 fl)	52.4	47.6	0.427
	Desirable (182–98 fl)	56.3	43.8	
	Above desirable (≥98.1 fl)	69.6	30.4	
Mean corpuscular hemoglobin	Below desirable (≤28.9 pg)	55.9	44.1	0.543
	Desirable (29–33 pg)	56.6	43.4	
	Above desirable (≥33.1 pg)	71.4	28.6	
Mean corpuscular hemoglobin concentration	Below desirable (≤31.9%)	70	30	0.304
	Desirable (32–35%)	55	45	
	Above desirable (≥35.1%)	60	40	
Red cell distribution width	Below desirable (≤9.9%)	100	0	0.39
	Desirable (10–15%)	58.9	41.1	
	Above desirable (≥15.1%)	50	50	
Leukocytes	Below desirable (≤4.3 × 10^9^/L)	33.3	66.7	0.274
	Desirable (4.4–11.3 × 10^9^/L)	59.6	40.4	
	Above desirable (≥11.4 × 10^9^/L)	54.8	45.2	
Neutrophils	Below desirable (≤44.9%)	80	20	0.018
	Desirable (45–70%)	67.8	32.2	
	Above desirable (≥70.1%)	49.6	50.4	
Lymphocytes	Below desirable (≤19.9%)	54.5	45.5	0.431
	Desirable (20–45%)	63.4	36.6	
	Above desirable (≥45.1%)	50	50	
Monocytes	Below desirable (≤1.9%)	66.7	33.3	0.051
	Desirable (2–12%)	54.5	45.5	
	Above desirable (≥12.1%)	80	20	
Basophils	Desirable (0–2%)	57.7	42.3	-
Neutrophils	Below desirable (≤1.79 × 10^9^/L)	50	50	0.257
	Desirable (1.8–7.7 × 10^9^/L)	61.3	38.7	
	Above desirable (≥7.71 × 10^9^/L)	50	50	
Lymphocytes	Below desirable (≤0.99 × 10^9^/L)	39.6	60.4	0.013
	Desirable (1.0–4.0 × 10^9^/L)	62.4	37.6	
	Above desirable (≥4.1 × 10^9^/L)	66.7	33.3	
Monocytes	Below desirable (≤0.19 × 10^9^/L)	0	100	0.216
	Desirable (0.2–0.8 × 10^9^/L)	56.3	43.8	
	Above desirable (≥0.81^9^/L)	60.5	39.5	
Eosinophils	Desirable (0–0.5 × 10^9^/L)	57	43	0.386
	Above desirable (≥0.51^9^/L)	100	0	
Basophils	Desirable (0–0.2 × 10^9^/L)	57.3	42.7	0.836
	Above desirable (≥0.21^9^/L)	50	50	
Platelets	Below desirable (≤129.99 × 10^9^/L)	72.7	27.3	0.045
	Desirable (130–450 × 10^9^/L)	56.7	43.3	
	Above desirable (≥450 × 10^9^/L)	16.7	83.3	
Mean platelet volume	Desirable (7.0–11.0 fl)	51.4	48.6	0.003
	Above desirable (≥11.1 fl)	74.1	25.9	
Neutrophil–lymphocyte ratio	Below desirable (≤0.99)	66.7	33.3	0.416
	Desirable (1–3)	64.2	35.8	
	Above desirable (≥3.1)	54.8	45.2	
Platelet–lymphocyte ratio	Below desirable (≤36)	-	-	0.031
	Desirable (36.1–172)	76.9	23.1	
	Above desirable (≥172.1)	54.6	45.4	
Coagulation				
Prothrombin time	Desirable (11–13.5 s)	64.3	35.7	0.251
	Above desirable (≥13.6 s)	56	44	
Quick index	Below desirable (≤74.9%)	55.9	44.1	0.596
	Desirable (75–120%)	59.7	40.3	
INR	Desirable (0–1)	63.6	36.4	0.421
	Above desirable (≥1.1)	57	43	
Activated partial thromboplastin time	Below desirable (≤19.9 s)	100	0	0.705
	Desirable (20–38 s)	59	41	
	Above desirable (≥38.1 s)	58.3	41.7	
Thromboplastin time ratio	Below desirable (≤0.89)	64.3	35.7	0.44
	Desirable (0.90–1.20)	55.6	44.4	
	Above desirable (≥1.21)	65.6	34.4	
Fibrinogen	Below desirable (≤149.9 mg/dL)	0	100	0.417
	Desirable (150–550 mg/dL)	61	39	
	Above desirable (≥550.1 mg/dL)	56.1	43.9	
Urine				
Density	Below desirable (≤1009.9 mg/mL)	75	25	0.954
	Desirable (1010–1030 mg/mL)	74	26	
	Above desirable (≥1030.1 mg/mL)	80	20	
pH	Desirable (5.0–9.0)	74.3	25.7	
Proteins	Desirable (≤10 mg/dL)	74.6	25.4	0.367
	Above desirable (>10 mg/dL)	73.1	26.9	
Urine glucose	Desirable (≤30 mg/dL)	74.6	25.4	0.876
	Above desirable (>30 mg/dL)	73.1	26.9	
Urine ketones	Desirable (Absent)	73	27	0.347
	Above desirable (Present)	38.3	16.7	
Urobilinogen	Desirable (≤1 mg/dL)	72.7	27.3	0.087
	Above desirable (>1 mg/dL)	100	0	
Urine erythrocytes	Desirable (≤10 cells/µL)	79.7	20.3	0.119
	Above desirable (>10 cells/µL)	68.2	31.8	
Density	Desirable (≤10 cells/µL)	80.3	19.7	0.1
	Above desirable (>10 cells/µL)	68.1	31.9	

**Table 4:** Two-factor model of the significant biomarkers in patient and controls according to the diagnosis.

	**C-Reactive Protein, mg/L**		**Urea, mg/dL**		**Lymphocytes, %**	
**Diagnoses**	**Case** **Mean (SD)**	**Control** **Mean (SD)**	**Case** **Mean (SD)**	**Control** **Mean (SD)**	**Case** **Mean (SD)**	**Control** **Mean (SD)**
Delirium (without secondary diagnosis)	37.67 (56.19)	-	51.38 (45.81)	-	17.58 (8.28)	-
Cardiac disorders	87.77 (98.5)	25.83 (42.91)	84.85 (55.07)	55.53 (19.05)	14.18 (7.49)	20.5 (9.35)
Stroke	-	6.63 (7.42)	-	44.75 (17.23)	-	20.70 (9.91)
Renal disorders	36.06 (41.81)	56.21 (90.02)	39.43 (15.36)	64.70 (47.11)	20.7 (10.54)	14.67 (8.21)
Respiratory disorders	17.99 (27.06)	104.93 (107.87) **	61.20 (27.45)	53.31 (18.23)	13.22 (3.1)	15.47 (9.67)
COVID-19	-	75.77 (49.91)	-	46	-	23.8 (10.38)
Trauma disorders	20.91 (27.86)	44.03 (44.66)	57.83 (22.9)	57.5 (18.17)	18.48 (9.53)	8.3 (2.68)
Digestive disorders	46.87 (65.91)	34.96 (88.09)	54.83 (22.90)	56.73 (28.61)	15.78 (6.86)	15.65 (13.2)
Neurological disorders	25.32 (37.96)	3.98 (5.56)	39.8 (10.34)	49.33 (42.52)	22.3 (11.34)	17.4 (7.1)
Fever	-	127.68 (93.87)	-	37.5 (10.6)	-	13.6 (10.6)
Anxiety	2.57	-	32	-	21.3	-
Sepsis	5.81 (7.72)	-	64.33 (35.1)	-	16.76 (14.5)	-
Hematological disorders	28.21 (10.44)	-	44.5 (38.89)	-	10.2 (9.47)	-
	**Neutrophils, %**		**Monocytes, %**		**Neutrophil–Lymphocyte Ratio**	
**Diagnoses**	**Case** **Mean (SD)**	**Control** **Mean (SD)**	**Case** **Mean (SD)**	**Control** **Mean (SD)**	**Case** **Mean (SD)**	**Control** **Mean (SD)**
Delirium (without secondary diagnosis)	68.26 (14.96)	-	8.83 (4.03)	-	5.54 (6.37)	-
Cardiac disorders	75.79 (10.38)	69.75 (10.38)	8.38 (2.87)	8.18 (2.02)	8.19 (6.37)	4.28 (2.48)
Stroke	-	71.35 (12.84)	-	5.97 (1.8)	-	5.1 (4.9)
Renal disorders	68.48 (11.45)	76.9 (10.13)	8.98 (3.04)	7.22 (2.81)	4.96 (4.13)	9.4 (12.73)
Respiratory disorders	77.12 (5.81)	74 (10.57)	7.52 (1.64)	8.16 (2.42)	6.42 (2.54)	7.94 (7.24)
COVID-19	-	67.07 (14.89)	-	8.4 (5.42)	-	3.67 (2.94)
Trauma disorders	71.53 (11.1)	84.2 (4.78)	8.7 (2.47)	6.93 (1.77)	5.23 (3.47)	9.22 (4.99)
Digestive disorders	72.6 (8.94)	75.22 (15.66)	10.76 (4.91)	6.9 (2.41)	5.43 (3.67)	10.4 (9.01)
Neurological disorders	60.35 (17.09)	73.4 (8.01)	10.44 (2.75)	6.9 (2.41)	3.39 (2.18)	5.04 (2.67)
Fever	-	76.25 (21.42)	-	9 (9.33)	-	9.5 (9.55)
Anxiety	67.8	-	7.8	-	3.12	-
Sepsis	73.97 (18.11)	-	8.37 (2.71)	-	7.76 (6.39)	-
Hematological disorders	80.75 (17.46)	-	8 (7.07)	-	15 (15.55)	-
	**Platelets, ×10^9^/L**		**Platelet–Lymphocyte Ratio**		**Mean Platelet Volume, fl**	
**Diagnoses**	**Case** **Mean (SD)**	**Control** **Mean (SD)**	**Case** **Mean (SD)**	**Control** **Mean (SD)**	**Case** **Mean (SD)**	**Control** **Mean (SD)**
Delirium (without secondary diagnosis)	217.12 (62.56)	-	302.83 (138.16)	-	10.73 (0.95)	-
Cardiac disorders	241.57 (64.42)	232.67 (76.93)	297.59 (120.83)	382.73 (156.91)	10.75 (1.12)	10.24 (0.77)
Stroke	-	203.75 (23.57)	-	450.1 (116.34)	-	10.3 (1.48)
Renal disorders	225.16 (90.34)	283.17 (131.39)	283.42 (193.34)	433.61 (305.7)	10.75 (1.16)	10.38 (1.09)
Respiratory disorders	176.6 (53.84)	270.13 (166.27)	271.92 (85.78)	404.51 (304.74)	10.82 (1.35)	10.17 (0.75)
COVID-19	-	241.67 (103.56)	-	494.87 (366.37)	-	10.33 (0.4)
Trauma disorders	240.17 (89.56)	230.5 (51.86)	387.43 (228.97)	363.65 (102.45)	10.34 (0.56)	11.07 (1.19)
Digestive disorders	132.33 (69.95)	246.75 (72.14)	261.12 (112.11)	411.44 (384.57)	12.54 (0.84)	10.66 (1.03)
Neurological disorders	253.25 (117.49)	230.17 (65.46)	346.03 (207.61)	372.16 (114.96)	10.13 (0.79)	10.5 (0.95)
Fever	-	116.5 (44.55)	-	511.67 (322.91)	-	10.9 (0.42)
Anxiety	132	-	220	-	11.1	-
Sepsis	224 (47.69)	-	305.87 (19.84)	-	11.37 (0.31)	-
Hematological disorders	335.5 (62.93)	-	340.21 (33.29)	-	11.35 (0.91)	-

** *p* < 0.001.

## Data Availability

Data are available on request from the authors.

## Supplementary Materials

The following supporting information can be downloaded at: https://www.mdpi.com/article/10.3390/life12081127/s1, Supplementary File S1: STROBE Statement—Checklist of items that should be included in reports of case-control studies.
